# Development of a Smartphone‐Enabled Hypertension and Diabetes Mellitus Management Package to Facilitate Evidence‐Based Care Delivery in Primary Healthcare Facilities in India: The mPower Heart Project

**DOI:** 10.1161/JAHA.116.004343

**Published:** 2016-12-21

**Authors:** Vamadevan S. Ajay, Devraj Jindal, Ambuj Roy, Vidya Venugopal, Rakshit Sharma, Abha Pawar, Sanjay Kinra, Nikhil Tandon, Dorairaj Prabhakaran

**Affiliations:** ^1^ Centre for Chronic Disease Control (CCDC)New DelhiIndia; ^2^ Centre for Control of Chronic Conditions (CCCC)Public Health Foundation of India (PHFI)GurgaonIndia; ^3^ Department of CardiologyCentre for Control of Chronic Conditions (CCCC)All India Institute of Medical SciencesNew DelhiIndia; ^4^ Department of Endocrinology & MetabolismCentre for Control of Chronic Conditions (CCCC)All India Institute of Medical SciencesNew DelhiIndia; ^5^ Department of Non‐Communicable Disease EpidemiologyCentre for Control of Chronic Conditions (CCCC)London School of Hygiene & Tropical MedicineLondonUnited Kingdom

**Keywords:** diabetes mellitus, high blood pressure, hypertension, mHealth, nurse, primary care, Quality and Outcomes, Health Services, Information Technology, Hypertension, Cardiovascular Disease

## Abstract

**Background:**

The high burden of undetected and undertreated hypertension and diabetes mellitus is a major health challenge worldwide. The mPower Heart Project aimed to develop and test a feasible and scalable intervention for hypertension and diabetes mellitus by task‐sharing with the use of a mobile phone–based clinical decision support system at Community Health Centers in Himachal Pradesh, India.

**Methods and Results:**

The development of the intervention and mobile phone–based clinical decision support system was carried out using mixed methods in five Community Health Centers. The intervention was subsequently evaluated using pre–post evaluation design. During intervention, a nurse care coordinator screened, examined, and entered patient parameters into mobile phone–based clinical decision support system to generate a prescription, which was vetted by a physician. The change in systolic blood pressure, diastolic blood pressure, and fasting plasma glucose (FPG) over 18 months of intervention was quantified using generalized estimating equations models. During intervention, 6797 participants were enrolled. Six thousand sixteen participants had hypertension (mean systolic blood pressure: 146.1 mm Hg, 95% CI: 145.7, 146.5; diastolic blood pressure: 89.52 mm Hg, 95% CI: 89.33, 89.72), of which 3152 (52%) subjects were newly detected. Similarly, 1516 participants had diabetes mellitus (mean FPG: 177.9 mg/dL, 95% CI: 175.8, 180.0), of which 450 (30%) subjects were newly detected. The changes in systolic blood pressure, diastolic blood pressure, and FPG observed at 18 months of follow‐up were −14.6 mm Hg (95% CI: −15.3, −13.8), −7.6 mm Hg (CI: −8.0, −7.2), and −50.0 mg/dL (95% CI: −54.6, −45.5), respectively, and were statistically significant even after adjusting for age, sex, and Community Health Center.

**Conclusions:**

A nurse‐facilitated, mobile phone–based clinical decision support system‐enabled intervention in primary care was associated with improvements in blood pressure and blood glucose control and has the potential to scale‐up in resource poor settings.

**Clinical Trial Registration:**

URL: https://www.clinicaltrials.gov. Unique identifiers: NCT01794052. Clinical Trial Registry—India: CTRI/2013/02/003412.

## Introduction

High blood pressure and diabetes mellitus are major contributors to cardiovascular disease burden, a leading cause of mortality worldwide and in India.^1^, ^2^ A systematic review estimated that among adults, 33.8% of urban and 27.6% of rural population have hypertension in India.^3^ Diabetes mellitus prevalence in India varies between 10.9% and 14.2% and 3.0% to 8.3%, respectively in urban and rural adults.^4^ Kearney et al estimated that the number of persons with hypertension in India will rise from 118.2 million in 2000 to 213.5 million in 2025, which constitutes an 80% rise.^5^ Similarly, the International Diabetes Federation estimates that diabetes mellitus is likely to increase from 69.2 to 123.5 million by 2040.^6^ Multiple studies have reported poor awareness, treatment, and control of these 2 conditions in India.^3^, ^7^, ^8^ For example, in India only a tenth of the rural population and one fifth of the urban population with hypertension have their blood pressure under control. Given these facts, achieving the global voluntary targets for noncommunicable diseases (NCDs) by 2025 will be challenging for India unless access to care is expanded through task shifting/sharing, greater use of technology, and evidence‐based care provision. We therefore developed and tested a feasible and scalable intervention for hypertension and diabetes mellitus care, incorporating task‐sharing and a mobile phone–based decision support software system (mDSS) at Community Health Centers (CHCs) in the state of Himachal Pradesh in India.

## Methods

The study employed a mixed methods approach to design the intervention, which was subsequently implemented and evaluated at 5 primary care facilities using both qualitative and quantitative methods.

The design phase included review of literature to identify barriers for delivering care for hypertension and diabetes mellitus in primary care settings in India. Interventions to improve the process of care, including mHealth‐based clinical decision‐support tools, were also reviewed. Concurrently, a needs‐assessment exercise was carried out at the selected health facilities through a Health Facility Assessment tool developed using the *Indian Public Health Standards for Community Health Centres*.^9^ The facility assessment covered the 4 domains: service delivery, personnel, equipment, and drugs in relation to capacity for hypertension and diabetes mellitus care. In addition to health facility assessment, in‐depth interviews with stakeholders in the healthcare organization (health administrators, doctors, and nurses) were carried out. Furthermore, observation of the functioning of the clinics and assessment of the records of the health facility were also made. We collated the inputs from literature review, needs assessment exercise, in‐depth interviews with stakeholders, and consultations with domain experts (cardiologists, endocrinologists, and health administrators) to design the intervention. These inputs were used for preparing training modules and mobile phone–based clinical decision‐support software tools (details of mDSS development is described in the Results section) for the healthcare team.

In order to implement the intervention, the Principal Investigators (D.P. and N.T.) made a formal presentation before the Government Officials of the Himachal Pradesh. The intervention, namely, “mPower Heart Project,” was approved by the state government. As per the advice of the State Government, Solan District was chosen for the project, considering its better roads and mobile phone connectivity in comparison to other districts. Subsequently, the Project Coordinator (VSA) attended a monthly meeting of the health department at the district headquarters, during which the Chief Medical Officer of the district introduced the project to the Block Medical Officers (Chief Medical Officer of the CHCs) in order to ensure full support and cooperation from the health system.

Prior to commencing the mPower Heart Project, the Medical Officers and nurses of the CHCs were trained to deliver the intervention, using the training modules, developed centrally by the investigators (D.P., N.T., A.R.) and other experts in the field. In addition, to support the delivery of the intervention, the district and state officials were regularly engaged to ensure availability of generic medicines recommended by the Indian Public Health Standards in the local pharmacies. During the evaluation phase of the mPower Heart Project intervention in 5 CHCs, the following outcomes were assessed:

1. Number of patients attending the outpatient clinic (Clinic) eligible for opportunistic screening.
2. Number of known cases of hypertension/diabetes mellitus attending the clinic.
3. Number of new cases of hypertension/diabetes mellitus detected through opportunistic screening.
4. The mean change in systolic blood pressure (SBP), diastolic blood pressure (DBP), and fasting plasma glucose (FPG) up to the 18 months of follow‐up.

Throughout the duration of the mPower Heart Project, participants were advised to come back to the CHCs at 3‐month intervals for follow‐up. Since the enrollment was a continuous process throughout the intervention period, the duration of follow‐up varied across participants. The longest follow‐up data (18 months) were available for those enrolled in the initial 3 months, while only baseline values were available for those subjects who enrolled toward the end of the intervention. We therefore decided to report the mean level of outcomes for every follow‐up until the 18th month of the project period, at which time point we had adequate follow‐up data. Hence, for evaluating outcomes such as SBP, DBP, and FPG, we considered 1 baseline visit and 6 follow‐ups (3, 6, 9, 12, 15, and 18 months of the project period) for the analysis.

### Statistical Analysis

Continuous variables are reported as means along with their confidence intervals. Categorical variables are reported as proportions. The change in SBP/DBP was examined among those with a diagnosis of hypertension or hypertension and diabetes mellitus, while the change in fasting blood glucose was studied among those comorbid with both conditions or a diagnosis of diabetes mellitus alone. Generalized estimating equations models with an unstructured correlation structure were used for the SBP analyses and an exchangeable correlation structure was used for the fasting blood glucose analyses.^10^ We used the Huber White sandwich estimator for variance in the fasting blood glucose models in order to address potential model misspecification.^11^, ^12^ The regression models included binary indicators for each follow‐up time point so as to quantify the change in SBP and FPG at each time point relative to the baseline. All models were adjusted for age, sex, and CHC in order to examine the change in clinical parameters after controlling of patient demographics. As part of a secondary analysis, we examined results from interaction models exploring the change in clinical parameters over time, comparing those with new versus known diagnoses of hypertension and/or diabetes mellitus. These models were also adjusted for patient demographics and CHC. We also fit similar interaction models between disease diagnosis groups (hypertension alone versus both hypertension and diabetes mellitus; diabetes mellitus alone versus hypertension and diabetes mellitus) and time in order to explore whether the change in the clinical parameters over time differed by disease diagnosis. All analyses were performed in STATA SE Version 13.

### Setting

The development and evaluation of the intervention was carried out in 5 government CHCs in the Solan district located in the north Indian state of Himachal Pradesh, India. The CHCs were situated in the blocks, namely, Dharampur, Dharlagarh, Syri, Kunihar, and Nallagarh. Each CHC catered to nearly 100 000 to 120 000 rural population. The CHCs were headed by a Block Medical Officer and had an additional 3 to 4 Medical Officers, all trained in modern medicine. The clinics at the CHCs catered to 80 to 200 patients each day.

### Timeline of Project

The project started with a design phase, which commenced in February 2012, seeking necessary approvals from the government. The evaluation of the intervention was carried out between December 1, 2012 and August 31, 2014. All participants gave written informed consent and the study was approved by the ethics committee of Centre for Chronic Disease Control (CCDC), New Delhi and has been registered with ClinicalTrials.gov (NCT01794052) and the Clinical Trial Registry of India (CTRI/2013/02/003412).

## Results

### Design of the Intervention

The health facility assessment and the in‐depth interviews provided rich feedback for designing the intervention. The critical gaps that were identified were insufficient personnel at the outpatient clinics of CHCs to provide care, lack of clarity/knowledge and subsequent insufficient use of clinical management guidelines, absence of educative tools to support and standardize patient care, insufficient supply of drugs, and lack of adequate laboratory support to guide therapy. Based on these inputs and consultations with experts, a 6‐component *“m‐Power Heart Project Intervention”* was designed that included the following:

1. A nurse care coordinator (NCC) to attend to individuals with hypertension and diabetes mellitus at the clinics to address the gap in personnel.
2. Structured training of the Medical Officers and NCC about the intervention.
3. Clinical management guideline for hypertension and diabetes mellitus (Tables S1 and S2).
4. A mobile phone–based clinical decision‐support software (mDSS) tool for the healthcare team to input patient demographics, history, and physical findings and to generate an individualized prescription based on standard clinical management guidelines.
5. Counseling services for patients on diet, tobacco, physical activity, and compliance with medicines.
6. A follow‐up plan for the patients to ensure long‐term care.

### Development of mDSS

**Figure 1:**
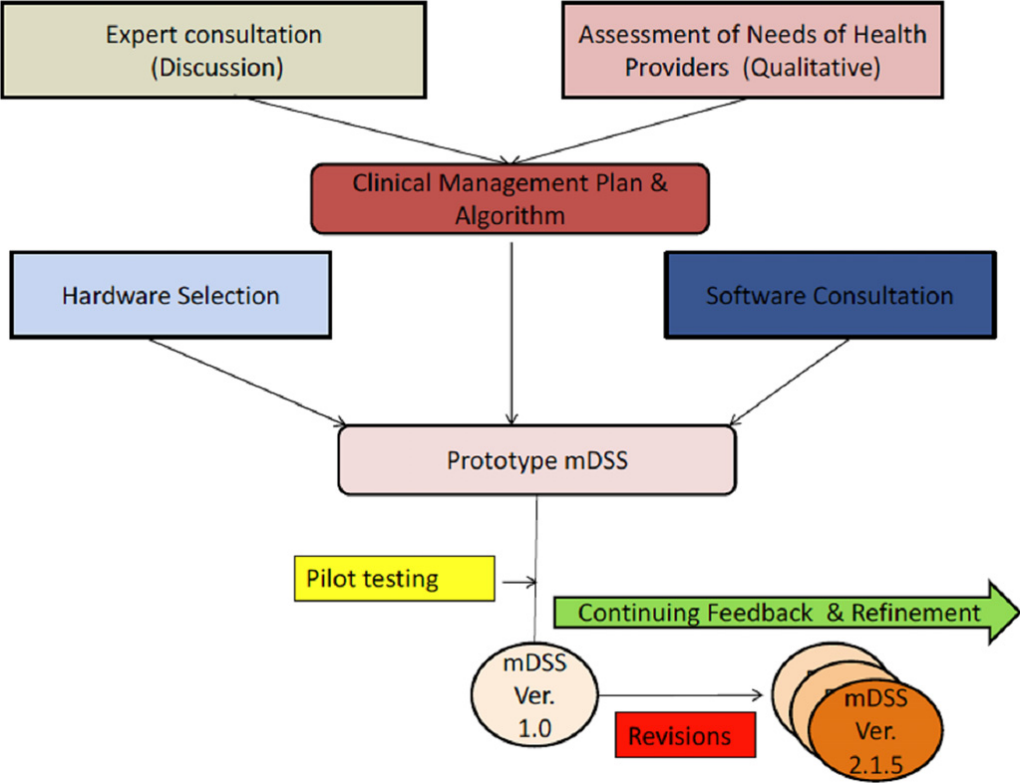
Steps in the development process of mDSS. mDSS indicates mobile phone–based clinical decision support system.

An expert group comprising cardiologists and endocrinologists designed a contextual clinical management guideline for hypertension and diabetes mellitus for the primary care physicians. Subsequently the guidelines were converted into mDSS by a software developer (Data Template Infotech Private Limited, Bangalore). The essential features of the mDSS (Table S3) and the design of the sequence of the user interfaces (Figure S1) were determined with the help of the developer and a prototype mDSS was developed. The mDSS initially required 36 data points for each patient to generate an individualized clinical management plan. The prototype was tested by developers by their in‐house software testing team for errors in software codes and logic given to them. The prototype was further tested by 2 researchers (V.S.A., D.J.) for the accuracy of the treatment plan generated using a range of possible scenarios. After fixing the errors, the prototype was field tested (user acceptance testing) with the help of two NCCs at the clinics for 2 weeks to elicit feedback on ease of use, interface and layout, and difficulties in comprehending inputs/outputs of the software. Incorporating this feedback, mDSS version 1.0 was released for deployment in 5 CHCs. At the end of 1 month, the NCCs reported that the Medical Officers may not co‐operate with them longer, because the high number of data elements required to be entered into the mDSS was chocking the patient flow. The expert group met again and decided to simplify the clinical management guideline. An updated mDSS (version 2.0) was released with 23 data points for the use of NCCs. Subsequently, 7 minor iterations were made to the mDSS, and the most recent version in use is version 2.1.5. A schematic diagram on the process followed in the development of mDSS is shown in Figure 1.

**Figure 2:**
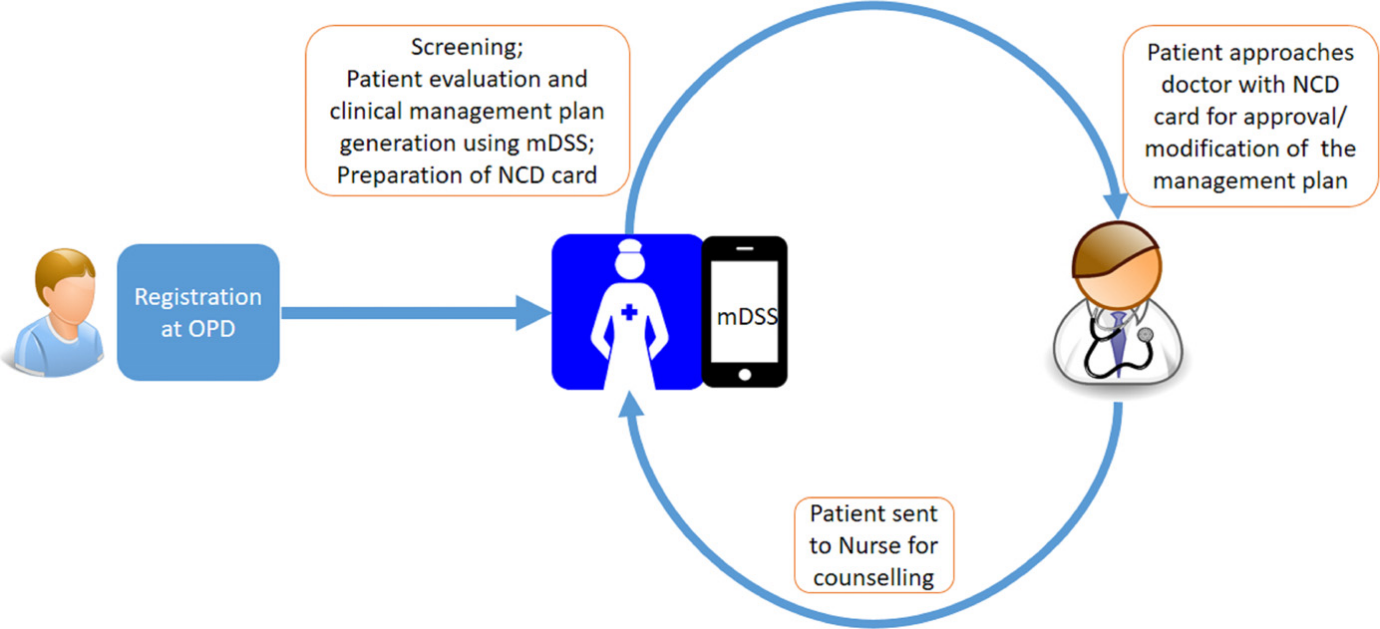
Workflow at the CHCs during the intervention. CHCs indicates Community Health Centers; mDSS mobile phone–based clinical decision support system; NCD, noncommunicable diseases; OPD, outpatient department.

The newly developed intervention was implemented in 5 CHCs with minor modifications in their workflow and was fully integrated into the routine care with least discomfort to the healthcare team or patients. Patients coming to the CHC first approached the registration counter where they received an outpatient card that recorded their name, age, and address. All patients above the age of 30 were directed to the NCC for screening of hypertension/diabetes mellitus. The NCC assessed their demographic details, medical history, anthropometry, and blood pressure. Additionally, fasting plasma glucose values were recorded for people with diabetes mellitus. Subsequently NCC fed this information into the mDSS tool to generate a personalized patient management plan. The mDSS‐generated patient management plan was then recorded onto a custom‐made NCD card and handed over to the patient. The patient then approached the medical officer along with the NCD card. The medical officer either approved the management plan or modified/rejected it according to his/her clinical judgment and recorded it in the NCD card with the reason for disagreement. The patient was then directed to the NCC, who then added the decisions of the doctor to the electronic patient record generated in the mDSS for future reference. The NCC then provided health education/counseling to the patients on drug intake, compliance, tobacco cessation, healthy diet, physical activity, moderation in alcohol use, and a follow‐up plan for future visits to the clinic (Figure 2).

Opportunistic screening resulted in 4 groups: (1) Screen positive subjects for hypertension; (2) Screen positive subjects for diabetes mellitus; (3) People with previous diagnosis of hypertension/diabetes mellitus or follow‐up cases; and (4) Screen negative subjects. At the time of opportunistic screening, screen positive hypertension subjects were advised to re‐visit for confirmatory diagnosis or put on hypertension management immediately (if blood pressure was >160/100) or referred to higher‐level centers (in case of hypertension urgency/emergency), depending on the blood pressure level and comorbidities. Screen positive diabetes mellitus subjects were advised to revisit the clinic in fasting state for confirmatory laboratory diagnosis. After confirmatory diagnosis of diabetes mellitus, the NCC generated a management plan using mDSS for the concurrence of the Medical Officer. People with known hypertension/diabetes mellitus or follow‐up cases visiting the CHC approached the NCC directly. Modification in their medication dosages were decided with the help of mDSS, which calculated optimal dose depending upon the clinical and laboratory values during that visit. People identified in the stage of prehypertension/prediabetes mellitus during screening were provided lifestyle counseling by the NCC.

### Effect of the Intervention on Outcome Indicators

After the complete rollout of the intervention on December 1, 2012 at 5 CHCs, a total of 132 370 patients attended these centers over the next 21 months until August 31, 2014. Of these, 22 009 (16.6%) subjects aged 30 years or above were eligible for opportunistic screening (Table 1). On screening the eligible group, 6797 (31%) subjects were identified to have either hypertension or diabetes mellitus, or both. Among these patients, nearly half (3391/6797) were diagnosed with hypertension/diabetes mellitus for the first time by opportunistic screening. Enrollment was a continuous process throughout the intervention period, and the duration of follow‐up varied across participants.

**Table 1:** Proportion of Known and New Subjects With Hypertension/Diabetes Mellitus Detected as a Result of Introducing Opportunistic Screening

Name of the CHC	Number of Patients Attended the CHC	Eligible Group for Screening	Subjects With Hypertension		Subjects With Diabetes Mellitus		Subjects With Both Hypertension & Diabetes Mellitus		% of Subjects With Hypertension or Diabetes Mellitus by Opportunistic Screening	
			Known	Newly Detected	Known	Newly Detected	Known	Newly Detected	Known	Newly Detected
Kunihar	34 633	4984	534	598	85	200	54	154	41.4	58.6
Syri	12 494	1824	173	330	12	28	8	37	32.8	67.2
Dharampur	28 564	3847	665	255	51	19	55	47	70.6	29.4
Dharlagarh	22 649	3533	414	353	24	22	14	24	53.1	46.9
Nalagarh	34 030	7821	1161	798	82	258	74	268	49.9	50.1
Total	132 370	22 009	2947	2334	254	527	205	530	50.1	49.9

CHC indicates Community Health Center.

### Impact on Blood Pressure Level Among Subjects With Hypertension

**Figure 3:**
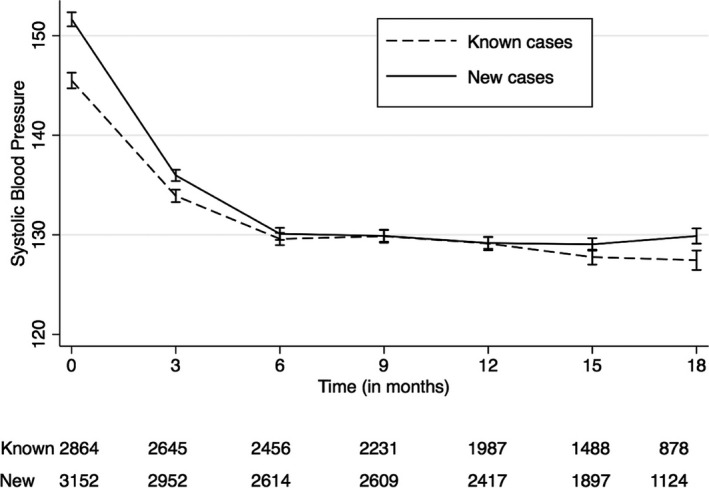
Change in mean systolic blood pressure level during 18 months of follow‐up; plotted with 95% confidence interval. Dashed black line: mean systolic blood pressure level among known cases during 18 months of follow‐up. Solid black line: mean systolic blood pressure level among newly diagnosed cases during 18 months of follow‐up.

A total of 6016 subjects with hypertension were enrolled, including 3152 (52%) newly detected by opportunistic screening. Among people with hypertension, the longest follow‐up data (18 months) were available for 759 subjects, while only baseline values were available for 292 subjects who enrolled towards the end of the intervention. The mean SBP at baseline was 146.1 mm Hg (95% CI: 145.7, 146.5), with newly diagnosed subjects having a higher SBP at baseline (149.1 mm Hg, 95% CI: 148.6, 149.7) as compared to subjects with known hypertension (143.0 mm Hg, 95% CI: 142.4, 143.6). During intervention, major reduction in SBP (−12.9 mm Hg, 95% CI: −13.2, −12.7) occurred during the initial 3 months of enrollment, which was sustained at 18 months (−14.6 mm Hg, 95% CI: −15.3, −13.8). Though the 2 groups reached similar blood pressure levels at 12 months, they tended to diverge at further follow‐up with higher mean SBP among newly diagnosed subjects as compared to people with known hypertension (Figure 3). The reductions observed in SBP levels in both of the groups at all the time points, in comparison with their baseline estimates, were statistically significant even after adjusting for age, sex, and CHC (Table 2) and were significantly higher among newly diagnosed subjects throughout 18 months of follow‐up.

**Table 2:** Change in Blood Pressure From Baseline to 18 Months

Time	Mean Change in SBP mm Hg (95% CI)				Mean Change in DBP mm Hg (95% CI)			
	Overall (Unadjusted)	Overall (Adjusted for Age, Sex, and CHC)	New Cases^a^	Known Cases^a^	Overall (Unadjusted)	Overall (Adjusted for Age, Sex, and CHC)	New Cases^a^	Known Cases^a^
3 months	−12.9^b^ (−13.2, −12.7)	−13.5^b^ (−13.7, −13.3)	−15.3^b^ (−15.6, −15.0)	−11.3^b^ (−11.6, −11.0)	−7.1^b^ (−7.3, −6.9)	−7.1^b^ (−7.2, −6.9)	−8.9^b^ (−9.2, −8.7)	−4.9^b^ (−5.2, −4.7)
6 months	−17.0^b^ (−17.5, −16.6)	−17.5^b^ (−17.9, −17.1)	−20.2^b^ (−20.7, −19.6)	−14.6^b^ (−15.2, −14.0)	−8.1^b^ (−8.4, −7.9)	−8.1^b^ (−8.3, −7.8)	−10.2^b^ (−10.6, −9.9)	−5.7^b^ (−6.1, −5.4)
9 months	−16.1^b^ (−16.6, −15.6)	−16.6^b^ (−17.1, −16.1)	−19.4^b^ (−20.1, −18.7)	−13.4^b^ (−14.1, −12.7)	−7.7^b^ (−8.0, −7.4)	−7.8^b^ (−7.9, −7.3)	−10.1^b^ (−10.4, −9.7)	−4.9^b^ (−5.3, −4.5)
12 months	−15.9^b^ (−16.5, −15.3)	−16.5^b^ (−17.1, −15.9)	−19.5^b^ (−20.3, −18.7)	−13.3^b^ (−14.2, −12.5)	−8.4^b^ (−8.7, −8.1)	−8.2^b^ (−8.5, −7.9)	−10.2^b^ (−10.6, −9.8)	−6.1^b^ (−6.5, −5.7)
15 months	−16.6^b^ (−17.3, −16.0)	−17.2^b^ (−17.8, −16.6)	−19.7^b^ (−20.5, −18.8)	−14.5^b^ (−15.5, −13.6)	−8.4^b^ (−8.7, −8.0)	−8.2^b^ (−8.6, −7.9)	−10.1^b^ (−10.5, −9.7)	−6.2^b^ (−6.7, −5.8)
18 months	−14.6^b^ (−15.3, −13.8)	−16.2^b^ (−17.0, −15.5)	−18.6^b^ (−19.6, −17.6)	−14.3^b^ (−15.4, −13.1)	−7.7^b^ (−8.0, −7.3)	−7.8^b^ (−8.2, −7.4)	−10.0^b^ (−10.6, −9.5)	−5.8^b^ (−6.3, −5.2)
Constant	146.1^b^ (145.7, 146.5)	135.9^b^ (135.1, 136.8)	132.4^b^ (131.4, 133.3)		89.5^b^ (89.3, 89.7)	86.5^b^ (86.1, 86.9)	86.5^b^ (86.1, 86.9)	
N	6016	6016	3152	2864	6016	6016	3152	2864

CHC indicates Community Health Center; DBP, diastolic blood pressure; SBP, systolic blood pressure.

a Results from the interaction model.

b *P*<0.001.

Our study had individuals with only hypertension (N=5281), only diabetes mellitus (N=781), as well as those comorbid with these conditions (N=735). The change in SBP level in both diagnoses groups (people with only hypertension versus those with hypertension and diabetes mellitus) were also statistically significant even after adjusting for age, sex, and CHC. However, the SBP change observed was significantly higher among people with hypertension alone until the ninth month of follow‐up, which tended to overlap during longer follow‐up (results not shown).

**Figure 4:**
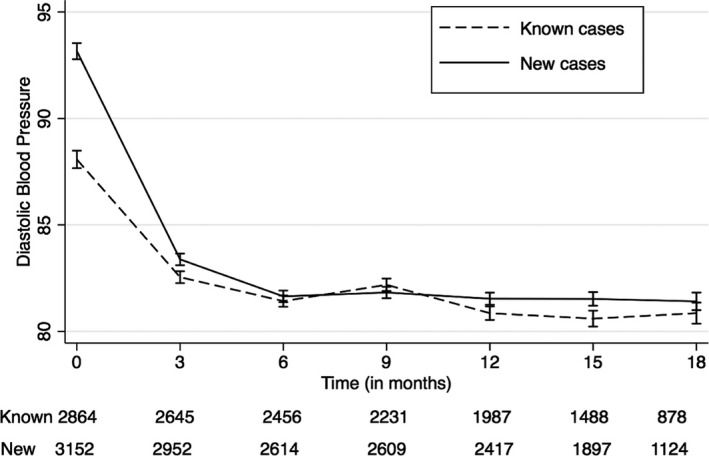
Change in mean diastolic blood pressure level during 18 months of follow‐up; plotted with 95% confidence interval. Dashed black line: mean diastolic blood pressure level among known cases during 18 months of follow‐up. Solid black line: mean diastolic blood pressure level among newly diagnosed cases during 18 months of follow‐up.

Similarly, statistically significant reduction in DBP (−7.1 mm Hg; 95% CI: −7.3, −6.9) occurred during the initial 3‐month period from a mean baseline level of 89.52 mm Hg (95% CI: 89.33, 89.72) with an overall change of −7.7 mm Hg (95% CI: −8.0, −7.3) at 18 months of follow‐up. Individuals who had newly diagnosed hypertension experienced a larger reduction in mean DBP (−10.2 mm Hg; 95% CI: −10.8, −9.7) compared to individuals with known hypertension (−5.6 mm Hg; 95% CI: −6.2, −5.0) at 18 months. The 2 groups reached similar blood pressure levels at 6‐month follow‐up and were similar at remaining follow‐up points (Figure 4). The reductions observed in DBP levels in both of the groups at all the time points, in comparison with their baseline estimates, were significant even after adjusting for age, sex, and CHC (Table 2) and were significantly higher among newly diagnosed subjects throughout 18 months of follow‐up. The change in DBP level in both diagnoses groups (people with hypertension alone versus those with both hypertension and diabetes mellitus) was statistically significant even after adjusting for age, sex, and CHC. In addition, the change in DBP was significantly higher among people with hypertension alone throughout the follow‐up (results not shown).

### Impact of the Intervention on Blood Glucose Levels of Diabetes Mellitus Patients

**Figure 5:**
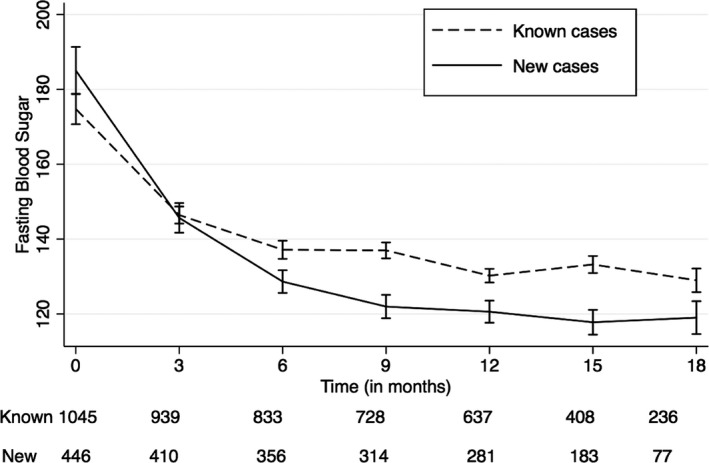
Change in mean fasting blood glucose level during 18 months of follow‐up; plotted with 95% confidence interval. Dashed black line: mean fasting blood glucose level among known cases during 18 months of follow‐up. Solid black line: mean fasting blood glucose level among newly diagnosed cases during 18 months of follow‐up.

A total of 1516 participants with diabetes mellitus were enrolled in the study, of which 450 (30%) subjects were newly detected by opportunistic screening. Among people with diabetes mellitus, longest follow‐up data were available for 293 subjects (18 months), while only baseline values were available for 114 subjects who enrolled toward the end of intervention. The mean fasting blood glucose at baseline was 177.9 mg/dL (95% CI: 175.8, 180.0), with the newly diagnosed subjects having a higher glucose level at baseline (185.1 mg/dL, 95% CI: 181.2, 188.9) as compared to subjects with known diabetes mellitus (174.8 mg/dL, 95% CI: 172.3, 177.3). The reduction in mean FPG level observed during 18 months of follow‐up was −50.2 mg/dL (95% CI: −54.7, −45.7) with major reduction occurring in the first 3 months of follow‐up. The reduction in FPG level was impressive and substantial in comparison to baseline levels even after adjusting for age, sex, and CHC (Table 3). Newly detected subjects with diabetes mellitus had a greater reduction in their mean FPG level (−63.7 mg/dL; 95% CI: −72.2, −55.3) than those with known disease (−44.7 mg/dL; 95% CI: −50.0, −39.4). The 2 groups varied significantly in their mean FPG level during the entire follow‐up period, except during their third month of follow‐up, with significantly higher mean FPG level among those known to have diabetes mellitus at recruitment (Figure 5). The 2 groups differed in their reduction in FPG level throughout the follow‐up, even after adjusting for age, sex, and CHC. The reduction in FPG level in both diagnoses groups (people with diabetes mellitus alone versus those with both hypertension and diabetes mellitus), when adjusted for age, sex, and CHC, was statistically significant, with higher reduction among people with diabetes mellitus alone throughout the follow‐up, except during third month of follow‐up (results not shown).

**Table 3:** Mean Change in Fasting Plasma Glucose Over Time mg/dL (95% CI)

Time	Overall (Unadjusted)	Overall (Adjusted for Age, Sex, and CHC)	New Cases^a^	Known Cases^a^
3 months	−31.5^b^ (−34.1, −28.9)	−27.4^b^ (−30.2, −24.6)	−36.1^b^ (−41.4, −30.7)	−24.0^b^ (−27.3, −20.7)
6 months	−42.5^b^ (−45.2, −39.8)	−38.0^b^ (−41.2, −34.9)	−51.9^b^ (−57.4, −46.3)	−32.4^b^ (−36.2, −28.6)
9 months	−44.6^b^ (−47.4, −41.7)	−39.7^b^ (−43.0, −.36.3)	−57.4^b^ (−63.4, −51.3)	−32.4^b^ (−36.3, −28.5)
12 months	−49.3^b^ (−52.0, −46.3)	−44.4^b^ (−47.8, −41.0)	−57.5^b^ (−63.6, −51.3)	−39.0^b^ (−43.0, −35.0)
15 months	−46.8^b^ (−50.3, −43.3)	−41.5^b^ (−45.1, −37.8)	−57.2^b^ (−63.5, −51.0)	−34.9^b^ (−39.2, −30.6)
18 months	−50.2^b^ (−54.7, −45.7)	−44.3^b^ (−48.3, −40.3)	−58.57^b^ (−65.7, −51.5)	−38.7^b^ (−43.4, −34.0)
Constant	177.9^b^ (175.8, 180.0)	182.1^b^ (176.2, 188.1)	175.5^b^ (169.2, 181.9)	
N	1516	1516	459	1057

CHC indicates Community Health Center.

a Results from the interaction model.

b *P*<0.001.

We also analyzed the pattern of missing data for all of the 5 CHCs. Given that participants could enroll at any time point throughout the intervention period, the individuals recruited in the first few months of the program had the most follow‐up data compared to those recruited during the later months. Intermittent missing data pattern was not observed, indicating overall good follow‐up and retention in the program. We also had limited covariate data on the participants in the study, and therefore it was reasonable to make these assumptions consistent with the generalized estimating equations model.

### Agreement With mDSS and Supply of Medicines

We analyzed 100 consecutive prescriptions from each CHC and found that 73% of them were in agreement with the mDSS suggestion. The major reason for deviation was unavailability of medicine at the pharmacies of CHCs. We also assessed the availability of medicines at the pharmacy, for December 2012 to June 2013 (atenolol: 9900; enalapril: 12 200; amlodipine: 3000; metformin: 30 700), and found that medicine supply was grossly insufficient. Hence, close to 80% of the patients were relying on out‐of‐pocket spending for medicines.

## Discussion

To the best of our knowledge, this study is the first published report from India about the development and evaluation of an mDSS‐enabled intervention through a NCC for hypertension and diabetes mellitus care in primary care settings. This work demonstrates the feasibility of task sharing of physician duties with an mDSS‐empowered NCC for management of hypertension and diabetes mellitus in current primary care settings of India. Furthermore, this strategy led to an impressive and significant reduction in blood pressure and blood glucose levels that persisted up to 18 months of follow‐up.

The core activities of the intervention were opportunistic screening and evidence‐based clinical management. The results from this study reinforce the concerns about a huge unmet need for hypertension and diabetes mellitus care in primary care settings. Close to a third of the outpatients above 30 years of age were detected with hypertension and/or diabetes mellitus. Half of these were newly diagnosed cases, which emphasizes the importance of opportunistic screening. It also opens a new window of opportunity for effective screening and management of hypertension and diabetes mellitus in primary care settings with innovative strategies that may be instrumental in surmounting the huge burden of cardiovascular diseases and its risk factors in the country.

The large reductions in systolic blood pressure observed in this study were similar to some of the previous studies that reported findings from nurse‐led interventions for patients with hypertension in the developed country settings.^13^, ^14^ Furthermore, our observations are consistent with other large multifactorial health system interventions (comprising patient registry, evidence‐based guidelines, quality performance metrics, medical assistant, and single pill combination therapy) experiments in the United States such as the Kaiser Permanente Northern California Hypertension program, which resulted in near doubling of hypertension control rates from 43.6% in 2001 to 87.1% in 2011.^15^ In India, a randomized trial of a computer‐based clinical decision support tool for hypertension care at primary care setting has been found to be efficacious and cost‐effective with an estimated reduction of 10.1 mm Hg of SBP in the intervention arm.^16^ Another recent community health worker–led trial of cardiovascular risk reduction intervention in rural India and Tibet also demonstrated a reduction of 11.8 mm Hg of SBP in the intervention arm in a setting where people were not accessing follow‐up care from primary care facilities, even after being identified to be at high risk of cardiovascular diseases.^17^

We observed impressive and substantial reduction in mean blood glucose levels among diabetes mellitus patients. The change observed was even higher than those reported in meta‐analysis of quality improvement strategy trials in diabetes mellitus care from developed countries.^18^, ^19^ The baseline FPG levels among the diabetes mellitus group were very high and this could be the reason for the large reduction in fasting glucose values. These results have important implications for low‐and‐middle income countries where primary care facilities seldom carry out an opportunistic screening program for hypertension and diabetes mellitus and often undertreat such patients due to lack of structured instructions or inadequate training and overcrowding. In addition, physicians often pay scant attention to advising patients on behavior modifications and compliance with medications that are essential for managing these diseases. This intervention has great potential for scalability in India as well where the national NCD program aims to establish a NCD clinic in CHCs, with the appointment of a Medical Officer, nurse, and counselor, which has high synergy to implement m‐Power Heart model intervention.

Though it is difficult to dissect out the effect of individual components of the intervention, qualitative exploration revealed a major role for additional personnel—a trained NCC deployed at the clinics—in the intervention. The new NCCs partially or fully addressed the personnel shortage at the clinics of the hospitals and had a high level of acceptance from Medical Officers as well as patients as caregivers.

The mDSS tool also had a major role in promoting evidence‐based practices at CHCs and helped in overcoming the “clinical inertia.” It helped the healthcare team to conduct comprehensive patient evaluation in a structured manner and also served as an electronic health record. The intervention also highlights the utility of technology in primary care settings for NCD risk factors management. Several studies carried out earlier in developed nations provide support to the utility of technology in improving health outcomes in diabetes mellitus and hypertension care.^20^, ^21^

There were certain limitations of this intervention. A randomized control would be the “gold standard” for evaluation. Given the previous evidences of benefits that have been noted both for task shifting by NCCs and the limited evidence on an internet technology–based decision support system, we believed a demonstration project would be the best design to demonstrate feasibility, potential for scale‐up, and mimic real‐world health systems. Furthermore, due to limited resources available for the study and insistence of the health authorities to involve all the CHCs (due to ethical reasons), no control arm was available for comparison. Given the volume of patients screened and enrolled in a routine clinic, there were practical difficulties in obtaining detailed information on potential confounders such as duration of prior medication use, types of medication and comorbidities, and integrating all such information into the DSS. Therefore, the findings could have resulted from some unmeasured confounding in the regression models. In addition, the observations on the interaction effect of disease groups and time should be interpreted with caution, because of the large differences in sample size between subgroups.

The limited number of CHCs involved in the development and evaluation of the intervention could be another drawback, as the developed intervention might require further customization or modification for scaling‐up in other Indian states and other international settings. Given our limited resources, we did not evaluate health outcomes such as stroke, myocardial infarction, or death. The major strength of our study is the NCC, as the Government of India in its action plan for NCDs has sanctioned a nurse to assist physicians at NCD clinics to be located at CHC. This study attests to the utility of those extra personnel. The second major strength is the low cost of developing the mDSS (data not shown).

## Conclusions

Our study demonstrates the feasibility of nurse‐facilitated, mDSS‐enabled intervention for hypertension and diabetes mellitus care in the primary care setting in India. Though the results from this study offer great potential for scale‐up in the public health system in developing countries, more research needs to be carried out to test its real‐world efficacy in randomized control trials.

## Sources of Funding

This research study was supported by the Medtronic Foundation and a Wellcome Trust Capacity Strengthening Strategic Award to the Public Health Foundation of India and a consortium of UK universities. The funders had no role in the study design and analysis.

## Disclosures

None.

## Supporting information


**Table S1.** Clinical Management Guideline for Hypertension
**Table S2.** Clinical Management Guideline for Diabetes Mellitus
**Table S3.** Essential Features of the mDSS
**Figure S1.** Sequence of user interfaces in the mDSS.
