# Supplementary material for: Development of a Smartphone‐Enabled Hypertension and Diabetes Mellitus Management Package to Facilitate Evidence‐Based Care Delivery in Primary Healthcare Facilities in India: The mPower Heart Project
Source: J Am Heart Assoc. 2016 Dec 21;5(12):e004343. doi: 10.1161/JAHA.116.004343 (PMC5210443; doi:10.1161/JAHA.116.004343)

SUPPLEMENTAL MATERIAL

Table S1. Clinical Management Guideline for hypertension

**Clinical parameters**	**If Age<55**	**If Age≥55**	**Messages**
if (SBP≥140<160) OR (DBP≥90<100)	Life Style Advices, If on medication, continue	Life Style Advices, If on medication, continue	
if ((SBP≥160) OR (DBP≥100)) AND (NO co-morbidities- COPD/ASTHMA, MYOCARDIAL INFARCTION, RENAL/LIVER FAILURE, PVD, HEART BLOCK, DIABETES)	1st: ACEi/ARB or CCB<br>2nd: ACEi/ARB + CCB or ACEi/ARB+Diuretic<br>3rd: ACEi/ARB + CCB + Diuretic	1st: CCB or Diuretics<br>2nd: ACEi/ARB + CCB or CCB + Diuretic<br>3rd: ACEi/ARB + CCB + Diuretic	If on medication, consider increasing the dose up or adding 2nd/3rd line drug.
if ((SBP≥160) OR (DBP ≥100)) AND (COPD/ASTHMA)	1st: CCB<br>2nd: CCB + ACEi/ARB or CCB + Diuretic<br>3rd: ACEi/ARB + CCB + Diuretic	1st: CCB<br>2nd: CCB + ACEi/ARB or CCB + Diuretic<br>3rd:ACEi/ARB + CCB + Diuretic	Avoid BB. If on medication, consider increasing the dose up or adding 2nd/3rd line drug.
if ((SBP≥140) OR (DBP ≥90)) AND (DIABETES)	1st: ACEI/ARB + ASA<br>2nd: ACEi/ARB + CCB or ACEi/ARB + Diuretic along with ASA<br>3rd: ACEi/ARB + CCB + Diuretic along with ASA<br>4th: Add Alpha Blocker or BB along with ASA	1st: CCB (PREFERRED) or ASA OR ACEi/ARB along with ASA<br>2nd: ACEi/ARB + CCB or ACEi/ARB + Diuretic along with ASA<br>3rd: ACEi/ARB + CCB + Diuretic along with ASA<br>4th: Add Alpha Blocker or BB along with ASA	If on medication, consider titrating the dose up or adding 2nd/3rd line drug. ASA Contraindicated if SBP>160 or DBP>100 or having gastritis or Aspirin Allergy
if ((SBP≥140) OR (DBP ≥90)) AND (MYOCARDIAL INFRACTION)	1st: BB + ASA<br>2nd: BB + CCB or BB + ACEi/ARB along with ASA<br>3rd: BB + ACEi/ARB + CCB/Diuretics along with ASA	1st: BB + ASA<br>2nd: BB + CCB or BB + ACEi/ARB along with ASA<br>3rd: BB + ACEi/ARB + CCB/Diuretics along with ASA	If on medication, consider titrating the dose up or adding 2nd/3rd line drug. ASA Contraindicated if SBP>160 or DBP>100 or having gastritis or Aspirin Allergy

Clinical parameters	If Age<55	If Age≥55	Messages
if ((SBP≥140) OR (DBP ≥90)) AND (RENAL/LIVER FAILURE {CREATININE> 3mg})	1st: CCB<br>2nd: CCB + Diuretic<br>3rd: ADD Alpha Blocker or BB or Other Diuretic	1st: CCB<br>2nd: CCB + Diuretic<br>3rd: ADD Alpha Blocker or BB or Other Diuretic	Avoid ACEi/ARB. If on medication, consider titrating the dose up or adding 2nd/3rd line drug.
if ((SBP≥140) OR (DBP ≥90)) AND ((PVD))	1st: CCB + ASA<br>2nd: CCB + ACEi/ARB or CCB + Diuretic along with ASA<br>3rd: CCB + ACEi/ARB + Diuretic along with ASA<br>4th: Add Alpha Blocker or Other Diuretic along with ASA	1st: CCB + ASA<br>2nd: CCB + ACEi/ARB or CCB + Diuretic along with ASA<br>3rd: CCB + ACEi/ARB + Diuretic along with ASA<br>4th: Add Alpha Blocker or Other Diuretic along with ASA	Avoid BB. If on medication, consider titrating the dose up or adding 2nd/3rd line drug. ASA Contraindicated if SBP>160 or DBP>100 or having gastritis or Aspirin Allergy
if ((SBP≥140) OR (DBP ≥90)) AND ((HEART BLOCK))	1st: ACEi/ARB<br>2nd: ACEi/ARB + CCB or ACEi/ARB + Diuretic<br>3rd: ACEi/ARB + CCB + Diuretic<br>4th: Add Alpha Blocker or Other Diuretic	1st: CCB (PREFERRED) OR ACEi/ARB<br>2nd:CCB + ACEi/ARB or CCB + Diuretic<br>3rd: CCB + ACEi/ARB + Diuretic<br>4th: Add Alpha Blocker or Other Diuretic	Verify Again. DILTIAZEM & VERAPAMIL & BB Contraindicared. If on medication, consider titrating the dose up or adding 2nd/3rd line drug.
if DBP≥130	Hypertensive emergency. Refer the patient immediately	Hypertensive emergency. Refer the patient immediately	

ACEi: Angiotensin Converting Enzyme Inhibitors; ARB: angiotensin receptor blockers; ASA: acetylsalicylic acid; BB: Beta Blockers; CCB: Calcium Channel blockers; COPD: Chronic Obstructive Pulmonary Disease; DBP: Diastolic Blood Pressure; PVD: Peripheral Vascular Disease; SBP: Systolic Blood Pressure

Table S2. Clinical Management Guideline for Diabetes Mellitus

**FAIR CONTROL, INSUFFICIENT CONTROL=**	**((FPG<110) AND (PP=200-300)) OR ((FPG =110 - 130) AND (PP=140-300)) OR (((FPG>130) AND (PP<=200))**
If (No OHA USE) AND (No INSULIN USE) AND BMI <= 23	1 Unit SU (5mg Glibenclamide OR 80mg Gliclazide)
If (No OHA USE) AND (No INSULIN USE) AND BMI > 23	500mg Metformin
If (1 or 2 OHA, NOT AT MAXIMUM DOSE is in USE) AND (NO INSULIN USE)	Consider 1 UNIT increment (SU { 5mg Glibenclamide, OR 80mg Gliclazide}, OR 500mg Metformin, OR 15mg Pioglitzone)
If (2 OHA AT MAXIMUM DOSE IS IN USE) AND (NO INSULIN USE)	ADD 3rd OHA (500mg Metformin OR SU{ 5mg Glibenclamide, OR 80mg Gliclazide} OR 15mg Pioglitazone)
If (3 OHA AT MAXIMUM DOSE IS IN USE) AND (NO INSULIN USE)	Start Insulin-Bedtime NPH Dose (10 units or 0.2U/kg/day). MESSAGE*
**POOR CONTROL, VERY POOR CONTROL=**	**((FPG>130) AND (PP=201-300)) OR ((FPG<110) AND (PP≥301)) OR ((FPG=110-130) AND (P≥301)) OR ((FPG>130) AND (PP≥301))**
If (No OHA USE) AND (No INSULIN USE) AND BMI <= 23	2 Unit SU (10mg Glibenclamide OR 160mg Gliclazide)
If (No OHA USE) AND (No INSULIN USE) AND BMI > 23	1000mg Metformin
If (1 or 2 OHA, NOT AT MAXIMUM DOSE is in USE) AND (NO INSULIN USE)	Consider 2 Unit increment (2 of the same or 2 different) (SU { 10mg Glibenclamide OR 160mg Gliclazide} OR 1000mg Metformin OR 30mg Pioglitzone)
If (2 OHA AT MAXIMUM DOSE IS IN USE) AND (NO INSULIN USE)	ADD 1 Unit addition of 3rd OHA (500mg Metformin OR SU { 5mg Glibenclamide OR 80mg Gliclazide} OR 15mg Pioglitazone)
If (3 OHA AT MAXIMUM DOSE IS IN USE) AND (NO INSULIN USE)	Insulin-Bedtime NPH Dose (10 units or 0.2U/kg/day). MESSAGE*
IF (INSULIN IN USE) AND (FPG<=70)	Reduce NPH dose. MESSAGE*
IF (INSULIN IN USE) AND (FPG>120)	Increase NPH dose up to .5U/kg/day. MESSAGE*

**MESSAGE: Review FPG after 1 week (Target FPG: 80-110). If current insulin dose is @ .5U/kg, add AM NPH (10 units or 0.2U/kg/day), Maximum NPH upto 20 Units only. If target not achieved, shift to PM Insulin two times daily*

BMI: Body Mass Index; FPG: Fasting Plasma glucose; NPH: Neutral Protamine Hagedorn; OHA: Oral Hypoglycaemic Agents; PP: Post Prandial glucose

Table S3. Essential Features of the mDSS

1.	Generation of electronic patient records in the mobile phone and in a central database.
2.	Clinical risk score for diagnosing diabetes
3.	Generation of personalized management plan for hypertension
4.	Generation of personalized management plan for diabetes
5.	Generation of serial data to provide continuity of care during follow-up visits
6.	Quality assurance checks
7.	Ability to export to statistic software packages for analysing temporal trends

Figure S1. Sequence of user interfaces in the mDSS.

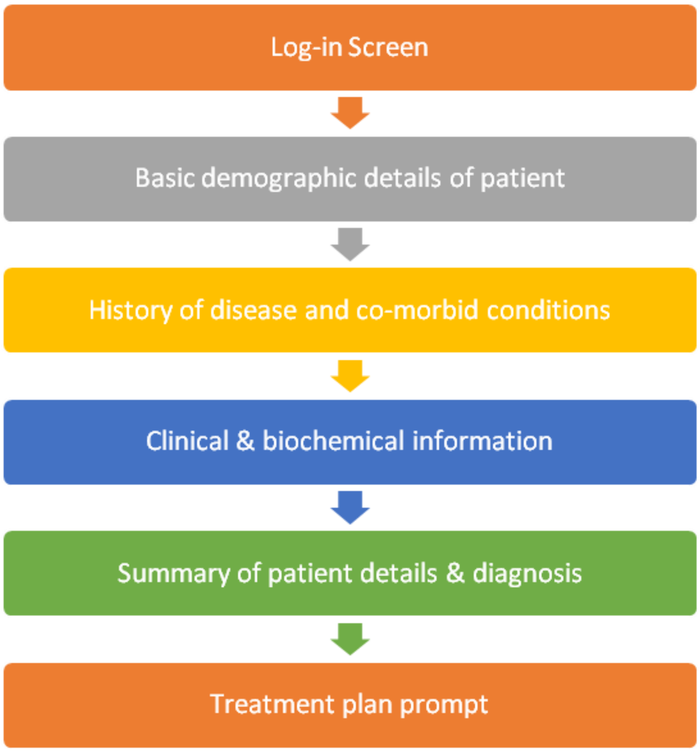

Supplement: Supplementary file 1 — Table S1. Clinical Management Guideline for Hypertension Table S2. Clinical Management Guideline for Diabetes Mellitus Table S3. Essential Features of the mDSS Figure S1. Sequence of user interfaces in the mDSS. [file JAH3-5-e004343-s001.pdf]
